# Annotations

**Published:** 1905-09-23

**Authors:** 


### ANNOTATIONS.

### Dr. Barnardo.

The somewhat sudden death, on Tuesday evening, of Dr. T. J. Barnardo, at the age of 60, is a distinct loss to the community. The founder of one of the most admirable of modern philanthropic projects, Dr. Barnardo was only 21 when his attention was called, as a student at the London Hospital, to the condition of the waifs and strays in the street—the children whom nobody cared for. He accordingly set himself to master the subject, and occupied his spare hours in studying it. In 1866 he first boarded out a number of children, and in 1867 he established his first Home. This was quite on a small scale, but the movement rapidly developed, because it had at its back a man with a strong personality, who combined marked originality with tenacity of purpose; and before his career was closed, to the infinite regret of all sorts of people, it could be said of him that he was the director of institutions by whose agency from 50,000 to 60,000 orphans have been received, trained, and placed out in life. Of these, a large proportion have emigrated to various British Colonies, and nothing afforded Dr. Barnardo greater pleasure than to read letters from them announcing their success. The Village for Girls at Ilford, and Her Majesty's Hospital for Sick Waifs, with nearly a hundred beds and a full staff, which owe their existence to him, subsequently along with numerous other developments divided his attention, but the original Home had always the first place in his regard. In 1891 he started the Young Helpers' League, and there is no doubt that by thus enlisting the personal enthusiasm of thousands of young people in his efforts he gave a powerful impetus to the flow of money in the direction of Stepney Causeway. He found time in his busy life for a good deal of literary work, but he wrote chiefly about children, and he himself described, as one of his recreations, "the society of children." It will be as a benefactor of children that he will be enduringly remembered, and no more splendid tribute could be possibly paid to his unremitting labours.

### Return Cases of Scarlet Fever and Diphtheria.

Not the least of the many causes of anxiety and worry to those who administer isolation hospitals is the not infrequent occurrence of subsequent infection of other individuals in the home of a patient who has been discharged from a scarlet fever or diphtheria ward. Such an event is nearly sure to be regarded by the man in the street as the result of carelessness on the part of the hospital authorities, for the general public, as represented by the daily press, are by no means gentle in their judgments of medical matters. Possibly this circumstance is a fortunate one; for once an evil comes to be regarded as inevitable its persistence is ensured. The report which has just been issued by the Metropolitan Asylums Board on return cases of scarlet fever and diphtheria shows how extremely difficult it is to avoid these cases. Dr. Cameron, who has drawn up the report after extensive inquiry and investigation, is only able to advise the adoption of certain experimental measures which may or may not be found to fulfil the conditions required. As to the pathology of these return cases, his most important conclusion is that there is a definite connection between the recrudescence of the disease and the existence in the discharged patient of an inflammatory condition of the nose or naso-pharynx; and it is also noted that in these cases there may be no recognisable evidence of such inflammatory condition at the time of the patient's discharge. Late desquamation, he says, cannot be looked upon as evidence of infectiousness. With regard to prevention of return cases, Dr. Cameron does not think this is by any means possible under the present conditions of hospital treatment. With extended means of isolation he thinks a reduction, perhaps a considerable reduction, in their number would result, and a bacteriological examination of both nose and throat of all cases of diphtheria prior to discharge would tend to reduce the number of the return cases of diphtheria. He also thinks it would be well, at any rate in the winter months, to omit the customary warm bath immediately before discharge, and to substitute one on the previous evening.

### Typhoid in Wells.

Attention was drawn on Tuesday by a correspondent of the *Times* to the ignorance which he alleges prevails among Local Government Board and London officials as well as urban and rural sanitary authorities concerning the rudiments of geological knowledge, by sanctioning schemes for sewage farms on and over highly porous strata. In support of his allegation he cites the case of the Clydach Valley in Breconshire and says that residents at the foot of the mountain have been drinking water from ancient springs, once pure, but now for years polluted by the carriers of the Local Government Board's authorised sewage farm of the town of Brynmawr. These sewage carriers, he says, leak and their foul contents sink into the crannies of the limestone rock, and poison the wells below, with the result that typhoid fever is scarcely ever absent from the little mountain valley. With regard to the danger of poisoned wells, a short time ago influenza, as it was called, appeared in a rural district in Cornwall, and it was not until the rector of the parish was attacked during a visit to London and the disease diagnosed in the metropolis, that it was found to be typhoid. A searching investigation on the spot was then made, and it was ascertained that the water in one of the farm wells had been polluted by sewage which had percolated through the ground some distance away. The rector himself had his own well, but the milk consumed in his house came from the farm, the cans being washed in the tainted water. It is easy to understand how this may happen in a remote parish, where the people do not possess even an elementary acquaintance with conditions of sanitation; but we agree with the correspondent of the *Times* that the Local Government Board, and, in fact, all sanitary authorities, ought to be, and no doubt are, fully aware that sewage-farms and sewage-carriers are necessarily sources of danger to the community if located in porous soil where water for drinking purposes is obtained from wells.

